# Evaluating the Efficacy of the “Support for Life” Program for People with Dementia and Their Families and Carers’ to Enable Them to Live Well: A Protocol for a Cluster Stepped Wedge Randomized Controlled Trial

**DOI:** 10.3389/fpubh.2016.00245

**Published:** 2016-10-31

**Authors:** Dianne Goeman, Tracy Comans, Joanne C. Enticott, Emma Renehan, Elizabeth Beattie, Susan Kurrle, Susan Koch

**Affiliations:** ^1^Royal District Nursing Service Ltd., RDNS Institute, St. Kilda, VIC, Australia; ^2^Faculty of Health and Medicine, The University of Newcastle, Newcastle, NSW, Australia; ^3^Metro North Hospital and Health Service District, Brisbane, QLD, Australia; ^4^Menzies Health Institute Queensland, Griffith University, Nathan, QLD, Australia; ^5^Synergy, Monash University, Dandenong, VIC, Australia; ^6^Dementia and Collaborative Research Centre (DCRC), School of Nursing, Queensland University of Technology, Brisbane, QLD, Australia; ^7^NHMRC Cognitive Decline Partnership Centre (CDPC), Sydney Medical School – Northern, Hornsby Ku-ring-gai Hospital, The University of Sydney, Sydney, NSW, Australia

**Keywords:** dementia, carer support, quality of life, well-being, RCT, protocol

## Abstract

**Introduction:**

Assistance provided to support people living with dementia and carers is highly valued by them. However, current support systems in Australia are disjointed, inaccessible to all, poorly coordinated, and focus on dysfunction rather than ability. Support workers for people with dementia are in short supply, and there is little consistency in their roles. To address this large service gap and unmet need, we have developed an evidence-based optimized model of holistic support for people with dementia and their carers and families. This article describes the “*Support for Life*” model intervention.

**Methods:**

A stepped wedge cluster randomized controlled trial will be conducted over 3 years across three Australian states. One hundred participants with dementia and/or their carers/family members will be randomly selected from community health center client lists in each state to receive either the dementia “*Support for Life*” intervention (Group A) or routine care (Group B). Group A participants will have access to the intervention from year 1. Group B participants will continue to receive usual care and will not be denied information on dementia or dementia services in year 1. In year 2, Group B participants will have access to the intervention. A highly trained expert dementia support worker will provide the “*Support for Life*” intervention, which is a flexible, individually tailored, holistic support that is relationship-centered, focused on enablement as opposed to dysfunction, and facilitate participants’ continued engagement in their community and the workforce. Additionally, dementia education, information resources, advocacy, and practical support to navigate and access dementia services and health care will be provided. The mode of support will include face to face, telephone, and internet interaction on an “as needed basis” for 12 months. The primary hypothesis is that the intervention will improve the quality of life of people with dementia and the health and well-being of carers/family through facilitating the continuation and enhancement of regular daily activities. Secondary hypotheses will examine other health and service usage outcomes. The outputs will also include a health economic analysis to investigate the costs (and savings) of any associated reduction in unnecessary health services use and delay in accessing permanent residential aged care.

**Trial registration number:**

Australian and New Zealand Clinical Trials Registry: ACTRN12616000927426p.

## Introduction: Background and Rationale

Models of support to assist people with dementia and their carers to adjust to living with memory loss, to navigate the health and aged care system, and to access services, information, and support have recently been implemented both in Australia and overseas. However, there has been little high level evaluation of these services prior to a recent project that evaluated the effectiveness of the support worker role through a systematic review of international literature of support workers and models of support workers operating across Australia (1, 2). This study was undertaken by members of the National Health and Medical Research Center (NHMRC) Cognitive Decline Partnership Center (CDPC) using a codesign approach involving consumers, industry partners, policy makers, and researchers to ensure a wide range of perspectives, including those of people with dementia, carer’s, and those working in the aged care industry (1, 2).

Findings from the CDPC project demonstrated that, although the dementia support worker role is highly valued, the current support system for people with dementia, their carers, and families is based on a biomedical model, is disjointed, poorly coordinated, and focuses on dysfunction and not on what people with dementia can still achieve. Additionally, no one service delivery model is accessible for people of all ages, diverse needs, or geographic locations. Our results also revealed insufficient numbers of support workers, support workers having high case loads that lead to them being time poor and experiencing burn out, and a lack of appropriate services available in the community to assist people with dementia and their families and carers to continue to undertake many of the regular activities of their daily life or actively engage in their community (2). Differences in available services were also dependent on the location of the service, such as the state in which the service was located and whether it was in a rural or metropolitan area (2). To address these gaps in services and the unmet needs of people with dementia and their carers and families, we have used the results from a systematic review of international models of support workers, an evaluation of Australian key worker models, and the perspectives of people with dementia and their carers to develop an optimized evidence-based support model (1–3).

Our evidence-based optimized dementia support worker model “*Support for Life*” is underpinned by an overarching philosophy that incorporates the four key themes: relationship-centered, enablement, holistic, and accessible. It articulates three components necessary to address current shortcomings: the necessary organizational context in which dementia support workers are able to operate autonomously, the definition of the role and what activities a dementia support worker role undertakes, and the competencies that are required to carry out the role successfully (2).

As no previous Australian studies have demonstrated, high level evidence of the efficacy of a consumer-directed dementia support worker model that focuses on enablement and meeting the individual needs of people with dementia and their carers/family members, we propose to test the efficacy of our optimized dementia support worker model by way of a cluster stepped wedge randomized controlled trial (4, 5).

This cluster stepped wedge randomized controlled trial will be the first Australian study to measure the efficacy and cost-effectiveness of the dementia support worker model. It is anticipated that this trial will provide high quality evidence of the efficacy and cost-effectiveness of a tailored, holistic, flexible, single service, and consistent approach to supporting people with dementia and their families and carers that promotes their well-being through the integration of services and health care, provision of emotional support, and enabling/encouraging them to remain actively engaged in the community.

### Explanation for Choice of Comparators

The design was developed to combine the rigor of a cluster randomized trial with the pragmatic approach of the stepped wedge design to implement the intervention at all sites (4–6).

### Objectives

To trial an optimized model, the “*Support for life*” program, based on the findings from the NHMRC CDPC evaluation that investigated the provision of support to people with dementia and their carers to address current shortcomings in support for people with dementia and their carers/families.

The “*Support for Life*” trial will be undertaken by a multidisciplinary team with extensive track records in all key areas of the project. This team combines expertise in dementia knowledge, translation knowledge, provision of support, and delivery of health services to people experiencing memory problems, cognitive impairment, or dementia; and research methods including randomized controlled trials (RCTs). The team will also have access to expert health economic, policy and research design through the CDPC enabling subunits, consumers and consumer representation through the Alzheimer’s Australia Consumer Dementia Research Network (CDRN) and statistical advice from the RDNS Institute statistician.

The “*Support for Life*” program is a whole of family approach to support that will assist people with dementia, their carers, and families to identify services that meet their individual need and offer them greater control and choice to continue to live and engage in life as fully as possible. This will help ensure that these services/supports include opportunities for people with dementia to have full access to “life activities” such as employment and recreation. Support will also focus on providing family members and/or carers the ability to actively participate in society and not be disadvantaged by their caring role. Active participation may mean continuing to work, attend school, university, and receiving appropriate support in these environments. To adhere to the overarching relationship-centered, enablement, holistic, and accessible principles that underpin the “*Support for Life*” program, workers providing support to people with dementia and their carers/families will

- require competencies in dementia knowledge, communication and interpersonal skills, the ability to generate ideas and problem solve, build and maintain relationships, and be empathetic and a good listener (1, 2).
- be enabled by a model of organizational support, which will ensure flexibility and autonomy, adequate resources, infrastructure and personal support, quality assurance practices, and facilitate appropriate inter-professional and inter-sectoral collaboration (1, 2).
- have a clearly defined description of the role that incorporates the flexibility and autonomy to refer and link to services, assist with navigation of the service system, act as a point of contact and advocate, respond to individual needs, provide dementia-related education and information, provide practical and emotional support (1, 2).

### Hypotheses

We hypothesize that connecting people with dementia and their families and carers to dementia support workers and volunteer mentors will lead to improvements in quality of life, reduce symptoms of depression, and facilitate the continuation of regular daily activities and enable community participation. The primary hypotheses are that involvement in the “*Support for life*” intervention will

- improve the quality of life of people with dementia in the intervention group compared with the control group.
- improve the health and well-being of carers and/or family members in the intervention group compared with the control group.

The secondary hypotheses are that the involvement in the “*Support for Life*” intervention will

- reduce depression experienced by people with dementia in the intervention group compared with the control group.
- reduce the utilization of health-care resources by people with dementia in the intervention group compared with the control group.
- lead to an increase in uptake of support/respite services/engagement in activities by people with dementia in the intervention group compared with the control group.
- lead to a delay in accessing permanent residential aged care by people with dementia in the intervention group compared with the control group.
- reduce depression/psychological distress experienced by carers and/or family members in the intervention group compared with the control group.

### Trial Design

The evaluation of the “*Support for Life*” complex intervention will occur within a step-wedge cluster Randomized Controlled Trial (cRCT) involving multiple community health centers across three states over 3 years. Clusters are the community health centers, and approximately 10 centers in each state will participate in this study. Half of the centers (Group A) will be randomized to receive the intervention in year 1 and the other half in year 2 (Group B).

This “*Support for Life*” complex intervention proposes to trial a framework of a single service, highly flexible holistic approach to support for people with dementia and their families/carers. It will provide a platform to integrate current services and health care, provide emotional support, and enhance community and social engagement. Important principles are

- Protecting the person with dementia and their rights to full citizenship regardless of their age, gender, and cultural background.
- Providing access to support where there is diminished decision-making capacity, retaining respect for the person with dementia but also the advocating for the rights of the family and caring unit.
- Improving access to palliation and “end of life care” in the setting of advanced or impaired decision-making, including with those who live alone.

This type of response builds on the strengths in a caring unit and addresses the needs of individual relationships. In line with “codesign” and “consumer directed care” (CDC) principles, the person with dementia, their family, and other members of the caring unit will actively design/influence what this service response will look like. Ongoing evaluation will allow for flexibility and change.

## Methods: Participants, Interventions, and Outcomes

### Study Setting

#### Community/Primary/Ambulatory Care

Collaborations with Primary Health Networks (PHNs) in Victoria, New South Wales, and Queensland will be used to assist with the identification of community health centers from metropolitan and regional areas, have a high incidence of patients with dementia, and diverse populations (including CALD and LGBTI).

### Eligibility Criteria

Criteria for inclusion will include people who are experiencing memory problems, cognitive decline, or having a formal diagnosis of dementia who are living in the community setting and willing to provide written, informed consent to participate in the study. People who are unable to self-manage but who have a carer will not be excluded from the study. Carers and family members of a person who is experiencing memory problems, cognitive decline, or dementia and are willing to provide written, informed consent are also eligible for participation in the study. The exclusion criteria include consumers who are already receiving a support intervention or have acute psychosis.

### Intervention

Dementia Support Workers, recruited from each of the three states, will inform the Community Health Centers of our trial of the provision of dementia friendly support to their patients by assisting them in getting the right services and the right people at the right place.

Group A participants will be from Group A cluster sites and will have access to the “*Support for Life*” intervention from year 1. This “*Support for Life*” complex intervention proposes to trial a framework of a single service, highly flexible holistic approach to support for people with dementia and their families/carers. It will provide a platform to integrate current services and health care, provide emotional support, and enhance community and social engagement by

- Working with the “person with dementia or cognitive impairment and those who support them” regardless of age, gender, and cultural background.
- Focusing on strengths of the people involved in order to maintain and improve physical and emotional health and well-being.
- Planning for the future to provide peace of mind.
- Use of an assistant and volunteers to help identify/create/facilitate access to services and enhance the social engagement of people with dementia or their carers and families in response to their stated goals or needs.
- Recognizing the challenges for young people and children and actively prototyping within the setting of their situation. It will require age appropriate responses and support mechanisms.

**Figure 1:**
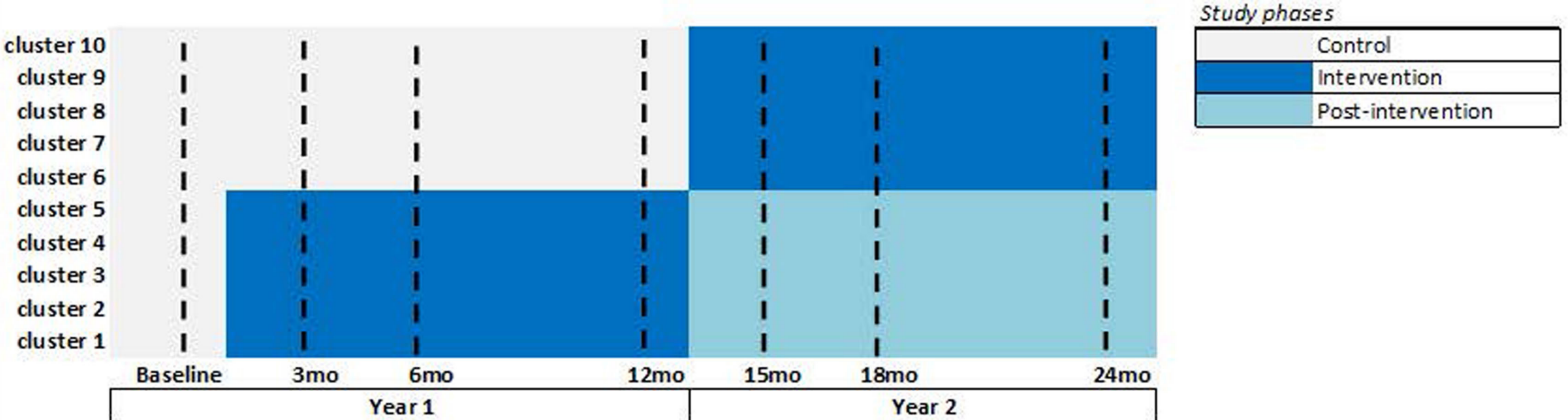
**“Support for Life” stepped wedge cluster randomized control trial (SWcRCT) study design**. Overall, there are 30 clusters participating, 10 clusters in each state, and so the above figure shows the design that is replicated in each state. In each cluster, we aim to recruit 10 study participants having dementia or cognitive impairment, making the overall recruitment target at 300 participants. Data are collected from all participants during the seven data collection time points shown by a dashed-line in the above figure (i.e., at baseline and 3, 6, 12, 15, 18, and 24 months). We expect an attrition rate of 20 percent; therefore, we expect approximately 240 participants with dementia or cognitive impairment and approximately 1,680 participant data points to be examined in this SWcRCT. Attrition is expected to occur at any time point, and the modeling will include all participant data; participant data may be complete (available from all seven time points) or incomplete (available from one to six time points). The intensive intervention phase is shown in dark blue. The post-intervention phase is shown in light-blue (this is the second year for the year 1 intervention group), where participants can still access the intervention, but the intervention will no longer be promoted at those clusters.

Group B participants will continue to receive usual care by health service providers and will not be denied information on dementia or dementia services in year 1. In year 2, Group B participants will have access to the “*Support for Life*” intervention making this a step-wedge study design. In year 2, Group A participants, who receive the intervention in year 1, may still access the intervention, but the intervention will no longer be actively promoted at those sites. See Figure 1 for the trial design.

### Outcomes

**Primary outcomes** (baseline, 3, 6, 12, 15, 18, and 24 months)
*Person with dementia*
  - Improvement in quality of life (suitable for use by people with moderate to severe levels of dementia) (QOL-AD) (7)
*Carer/family members*
  - Improvement in well-bein*g* of carer/family unit [Carer experience scale (CES)] (8, 9)
**Secondary outcomes** (baseline, 6, 12, 18, and 24 months)
*Person with dementia*
  - Reduction in symptoms of depression (GDS 15) (10)
  - Increased attendance at social activities/services (staff/family/carer report/interviews)
  - Increased engagement in social activities/services/ability to continue to participate in regular activities (staff/family/carer report/interviews)
  - Change in health-care service utilization [client records/resource utilization in dementia (RUD) (11) health records]
  - Delay in accessing permanent residential aged care services (client records/interviews)
*Carer/family members*
  - Reduction in carer depression/psychological distress [General Health Questionnaire-12 (GHQ-12)] (12)
  - Increase in social engagement and ability to continue regular activities (self report/interviews).

(See Table 1 for a full list of instruments).

**Table 1:** **Outcome measures**.

	Baseline	3 months	6 months	12 months	15 months	18 months	24 months
**Primary outcomes**							
Person with dementia: improvement in quality of life	QOL-AD	QOL-AD	QOL-AD	QOL-AD	QOL-AD	QOL-AD	QOL-AD
Carer/family members: improvement in well-being	CES	CES	CES	CES	CES	CES	CES
**Secondary outcomes**							
Person with dementia: reduced symptoms of depression	GDS 15		GDS 15			GDS 15	GDS 15
Person with dementia: increase in social activities	Interview/client records		Interview/client records	Interview/client records		Interview/client records	Interview/client records
Person with dementia: increase in social engagement	Interview/client records		Interview/client records	Interview/client records		Interview/client records	Interview/client records
Person with dementia: change in health service utilization	RUD		RUD	RUD		RUD	RUD
Person with dementia: delay in accessing permanent residential aged care services	Interview/client records		Interview/client records	Interview/client records		Interview/client records	Interview/client records
Carer/family members: reduction in depression/psychological distress	GHQ-12		GHQ-12			GHQ-12	GHQ-12
Carer/family members: increase in social engagement/ability to continue regular activities	Interview/client records		Interview/client records	Interview/client records		Interview/client records	Interview/client records

## Economic Methodology and Analysis

- Utility measured by EQ-5D-5L (13)
- Reduction in health service utilization measured by RUD and administrative data
- Reduced carer time (paid and unpaid) measured by RUD
- Delay in permanent admission to residential care.

### Schedule of Events

At baseline, we will administer the primary and secondary measures to all participants. At 3, 6, 12, 15, 18, and 24 months, we will administer the primary measures. At 6, 12, 18, and 24 months, we also administer the secondary measures. All participants will follow this schedule of data collection regardless of group allocation. See Figure 1.

The intensive intervention phase at each cluster is shown in Figure 1. During this phase, the intervention will be actively promoted at the indicated clusters. The post-intervention phase is shown in light-blue, where participants can still access the intervention, but the intervention will no longer be actively promoted at those clusters.

### Sample Size

The study is powered to detect small–medium effects in the primary outcome measures of QOL-AD for people with dementia or cognitive impairment (0.23 effect size) and in the CES for carers (0.25 effect size).

The sample size required is 30 clusters with 10 participants/families in each to allow for an attrition of 20%. This is a total of 100 participant/families (50 in each group) in each of the 3 states, making a total sample size of 300. This will detect significant differences in caregiver’s and people with dementia or cognitive impairment’s quality of life between two groups at effect sizes of 0.23–0.25 (small–medium effects). Sample size calculations were completed using STATA statistical software stepped wedge: for clusters defined at the level of the community health center, power of 0.80, significance level set at 0.05, intraclass correlation coefficient (ICC) of 0.05, the number of steps (2), participant data collected at seven time points (baseline, 3, 6, 12, 15, 18, and 24 months), and primary outcome distributions as described in existing literature, i.e., QOL-AD scores between 15 and 60 and SD of 7 (6, 7, 14, 15) and CES scores between 0 and 100 and SD of 10 (8, 9).

### Recruitment

Participants with dementia or cognitive impairment and their carers/family members will be randomly assigned from community health center client lists and then randomly allocated to receive either the dementia “Support for Life” program (Group A, *n* = 50 per state) or routine care only (Group B, *n* = 50 per state).

We will apply quota sampling so that clusters comprise of essentially similar participants. Ten participants from each cluster are required, and so recruitment will cease within the following subgroups at each cluster when the following characteristics are attained:

– Age
  ○ Five participants are aged <65 years
  ○ Five participants are aged >65 years
– Gender
  ○ Five females
  ○ Five males
– Diversity/cultural background
  ○ Eight born in Australia
  ○ Two from CALD/LGBTI communities

## Methods: Assignment of Interventions (For Controlled Trials)

### Randomization

Clusters are community health centers participating in the study. Randomization of clusters to an intervention year (1 or 2) will be done using a minimization routine balancing numbers in groups as randomization proceeds (16). To guard against confounding by major differences in clusters, randomization will be stratified by state (Victoria, New South Wales, or Queensland), area (metropolitan or regional), and socioeconomic status of area that cluster is located within [defined by 1–3 versus 4–5 quintile of the area index of relative disadvantage (IRSD)].

### Allocation

#### Sequence Generation

Participants with dementia or cognitive impairment and their carers/family members will be randomly selected from community health center client lists and then randomly selected to receive either the dementia “Support for Life” program (Group A, *n* = 50 per state) or routine care only (Group B, *n* = 50 per state).

#### Allocation Concealment Mechanism

Computer generated randomization of Group A and Group B will be performed by an independent researcher after the baseline data are collected.

#### Implementation

The allocation sequence will be developed by a statistician blinded to the study details. Identified potential participants will be approached, recruited, and enrolled by experienced members of the research team in each state. The statistician who generates the allocation sequence mentioned above will pass this code to the overseer of the recruitment process.

#### Blinding (Masking)

The Research Officer undertaking the data collection will be blinded to participant group allocation. Participants (people living with dementia or cognitive impairment in the community, carers of people with dementia or cognitive impairment living in the community, and family members of people with dementia or cognitive impairment living in the community) will be blinded to their group allocation.

## Methods: Data Collection, Management, and Analysis

Primary and secondary data collection will occur at baseline and 3, 6, 12, 15, 18, and 24 months post-randomization (Figure 1). This will involve a face to face visit with a researcher. Participants will receive a telephone call and an information letter with study questions 10 to 14 days prior to the baseline and all follow-up visits. The telephone contact will be used to arrange a mutually suitable time and location for the face to face visit as well as provide information to participants about what will take place at the forthcoming visit. Participants will have the option to complete questionnaires prior to the visit or complete them during the researchers visit. Interviews will take place during the face to face visit and will be audio-taped prior to transcription and importation into the NVivo Qualitative Software to aid analysis. The researcher will be trained in quantitative and qualitative data collection methods and any relevant outcome measure assessments. They will also be experienced in communicating with people with dementia and their carers and families including collecting data from participant and proxy (carer/family member) when necessary.

### Inter-Rater Reliability

To ensure consistency of the interpretation/coding of the qualitative interview data and the measurement of the quantitative data, we will calculate the correlation between the ratings of two researchers. Data coded into the statistical software will also be checked to ensure its accuracy.

### Data Management

All hard copy and audio-recorded data will be kept confidential and stored in a locked filing cabinet in the research group office and in softcopy in a password protected database according to ethical storage and disposal of data as stated in the NHMRC guidelines. Data will be only accessible to the chief investigator research officer, project manager, and statistician. The research team will monitor the integrity of the trial data. All participant data will be entered into an appropriate database such as Redcap (https://projectredcap.org/) or the Statistical Package for Social Sciences (SPSS) package and screened to detect any errors. A range of checks will be undertaken prior to data analysis.

The project manager will be employed to oversee the project and to manage the project data from each of the three states, including data cleaning and data merging.

### Statistical Methods

#### Data Analysis Plan

##### Quantitative Analysis

###### Primary Analyses

We will use all data along with the original designated group allocation and an intention to treat approach as the primary analytic framework. Descriptive data on both groups will be reported including demographics, stratification variables, baseline measures, and number of times intervention was accessed. Descriptive statistics will be used to summarize the characteristics of all participants including carers and family members. Deterioration of the primary outcome measure in individuals due to increasing dementia severity/aging and occurring over the 2-year data collection period will be explored using multivariate analyses (including factors: intervention status, age, and gender). If the dementia severity/aging factor is found to be significant, it will be included in the main models described below.

A linear mixed effects model will be used to compare the intervention and usual care periods for continuous outcomes and generalized linear mixed effects model for binary outcomes. The model will include intervention status and time as fixed effects and cluster site and individuals as random effects. Where appropriate, cluster and individual factors strongly correlated with the outcome will also be included as fixed effects in the model. These may include the stratification variables, e.g., socioeconomic status of area. The estimated intervention effect will be reported as the mean outcome difference for continuous outcomes and odds ratio for binary outcomes between intervention and control periods, assuming a constant treatment effect over time. The estimated intervention effects will be reported with 95% CIs and *p*-values. The analysis will investigate an interaction effect between intervention and time.

### Missing Data

The analysis method chosen will use all available data. Every effort will be made to minimize missing outcome data at each wave, and reasons individuals are lost to follow-up will be recorded. A missing data analysis will seek to identify systematic patterns of missing data and identify instances where, despite the efforts of the research team, data may be missing not at random (MNAR). Whether or not data are identified as MNAR, for each primary outcome and other key variables with missing data, multiple imputation using multivariate regression with factors of age, gender, state, and time will produce 100 estimates for each missing item – supporting optimal statistical estimation. Sensitivity analyses will be conducted to assess the robustness of the missing data assumption made in the primary analysis. A detailed analysis plan will be developed for secondary and sensitivity analyses.

#### Qualitative Analysis

Interview transcripts and journal entries will be managed using QSR NVivo (Qualitative Solutions and Research, Melbourne, Victoria). Interview data will be initially coded into broad topic areas using content analysis, and then constant comparative method will be applied to develop theoretical insights.

#### Economic Analysis

##### Resource Use

The cost of the delivery of the program will be calculated using actual wage rates, and resources used for each participant subtracting any resources used specifically for the purposes of research. Health-care resource use will be costed for each participant using administrative data or data collected using the RUD instrument by multiplying use by appropriate unit costs sourced from government databases. These include Medicare for items such as GP and specialist visits, PBS for medications, and National Hospital Cost data reports for hospital and ED presentations. Community care will be costed using administrative data from the RDNS. Costs over 12 months will be compared between groups using a generalized linear model, which is able to account for the non-normal distribution of cost data. Cost of carer time will be accounted for using a proxy good method (17) to recognize the value of unpaid caregiving duties. Costs will be reported by sector (health, community, and type of participant) in order to understand the implications of the intervention to different interest groups and sectors.

## Outcomes

The primary outcome for the economic analysis is the quality adjusted life years (QALYs). Utility values will be derived from the CES, and EQ-5D-5L will be used to construct QALYs over 12 months using an area under the curve analysis. QALYs for both the carer and the participant will be combined for use in a cost–utility analysis. Research is currently underway to develop an algorithm for estimating utility values from the QOL-AD (CI Comans), and if an algorithm is available, QALYs will also be estimated from this instrument and compared with the values obtained from the generic EQ-5D-5L instrument.

Cost and QALYs will be combined into an Incremental Cost-effectiveness ratio (ICER). The analysis will be over 12 months to match the trial duration and will take a limited societal perspective in order to incorporate the costs and outcomes of the carers and participants as well as the health-care costs and outcomes. Uncertainty in the cost–utility results will be estimated using non-parametric bootstrapping techniques and represented as cost-effectiveness acceptability curves.

## Methods: Monitoring Fidelity of the Intervention/Adherence to the Protocol

An advisory and data monitoring committee (ADMC) will be established for the study and a protocol prepared to provide guidance to the study research team. The role of the ADMC will be to advise investigators in regard to implementation, maintenance, and monitoring of the overall conduct of the trial; safeguard the interests of study participants; and assess the intervention to ensure any potential adverse events are averted. Membership of the advisory committee will include a geriatrician, senior clinical nurse advisor dementia, state government representatives, consumer advocacy groups, and consumers.

Audits to monitor the fidelity of the intervention and adherence to the protocol will be undertaken on a six monthly basis.

## Ethics and Dissemination

The Support for Life study involves working with participants who are living with memory loss or dementia and their carers and families. To ensure that the needs of these participants are met, the research team includes investigators with expert knowledge in dementia and aged care services.

Approval to conduct the study has been applied for from the RDNS Human Research Ethics Committee in Victoria and will also be sought from The University of Sydney Human Research Ethics Committee in New South Wales and the Queensland University of Technology Human Research Ethics Committee in Queensland. All participants will be provided with detailed written information and have an opportunity to discuss participation before providing written or verbal consent. Verbal consent will be audio-taped. Participants will be advised that they can withdraw from the study at any point.

Any required modifications to the study protocol, such as changes to eligibility criteria, outcomes, and analyses, will be discussed by the study investigators and after agreement is reached will be submitted to the abovementioned Ethics committees for approval. Amendments describing any modifications to the study protocol will also be made to the relevant trials registry.

### Confidentiality

Personal information about potential and enrolled participants will be collected and then de-identified prior to it being shared to ensure confidentiality of participants is maintained before, during, and after the trial. Only the project manager, research team, and dementia support worker will have access to the final trial dataset.

### Declaration of Interests

The study investigators declare that they have no conflicts of interest.

### Access to Data

Only investigators and approved researchers added by ethics approval will have access to the final trial data set.

### Ancillary and Post Trial Care

The intervention has been developed by a lead service provider and a research team with expertise in dementia care, dementia research, and interventions for people with dementia, it is believed that the need to discontinue the intervention will be extremely minimal. If any participant becomes distressed as a result of participation in our trial, they will be referred to appropriate counseling support services.

### Dissemination Policy

Results from the trial will be provided to study participants and disseminated to health-care professionals through presentation at seminars, conferences, and publication in scientific journals and relevant media. Guidelines for authorship will be adhered to.

### Appendices

#### Informed Consent Materials

Model consent form and other related documentation will be given to participants and authorized surrogates.

## Strengths and Limitations of This Study

- This study is the first to implement a stepped wedge cluster randomized controlled trial (SWCRCT) design to provide high quality evidence of the efficacy and cost-effectiveness of a tailored, holistic, flexible, single service, and consistent model that promotes the well-being of people with cognitive impairment or dementia, their carers, and their family and enables/encourages them to remain actively engaged in the community.
- A major strength is that the development of the intervention development used high quality evidence gained from a systematic review of international models of support workers; an evaluation of Australian key worker models; and a codesign approach with people with dementia and aged care industry partners, policy makers, and researchers.
- With the stepped wedge design, all clusters will ultimately receive the intervention, while those waiting for the intervention to commence act as controls.
- The stepped wedge design means that some clusters wait for up to 12 months before starting the intervention, which may increase dropout rates and decrease motivation for participation.
- The stepped wedge design will overcome the logistical constraint of not being able to deliver the intervention concurrently to all clusters.
- Using a stepped wedge design will also enable all participating clusters to ultimately receive the “Support for Life” intervention, which is an advantage when working with a vulnerable population group where it is not ethical to withhold an intervention that is perceived to be beneficial.

## Author Contributions

SKO and DG conceived the study. The CDPC Activity 3, 4, and 17 research teams, working group, and reference group members contributed to the development and refinement of the proposal in conjunction with DG, SKO, ER, SK, and EB. JE led the calculation of the sample size and quantitative components of the protocol, and TC led the economic evaluation components. DG drafted the protocol paper, and all the authors contributed to the draft and approved the final version of the paper.

## Conflict of Interest Statement

The authors declare that the research was conducted in the absence of any commercial or financial relationships that could be construed as a potential conflict of interest.
